# EHMTI-0333. The prevalence and burden of migraine in india: results of a population-based study in Karnataka state

**DOI:** 10.1186/1129-2377-15-S1-B18

**Published:** 2014-09-18

**Authors:** G Kulkarni, G Rao, G Gururaj, DK Subbakrishna, T Steiner, LJ Stovner

**Affiliations:** 1 Neurology, National Institute of Mental health & Neurosciences, Bangalore, India; 2 Epidemiology, National Institute of Mental Health & Neurosciences, Bangalore, India; 3 Bio-statistics, National Institute of Mental Health & Neurosciences, Bangalore, India; 4 Neurosciences, Imperial College, London, UK; 5 Neurosciences, Norwegian University of Science and Technology, Trondheim, Norway

## Background

Migraine is the 3rd most prevalent and 7th leading cause of disability worldwide. India, where the prevalence of migraine is unknown, is the 2nd most populous country in the world.

## Aim

To estimate the prevalence and disability burden attributed to migraine in Karnataka, South India.

## Methods

Ethics approval and informed consent from participants were obtained. Trained interviewers selected households by random cluster sampling in urban (n=1,226) and rural (n=1,103) populations. They called unannounced at each and interviewed one adult randomly per household using a modified HARDSHIP questionnaire. Migraine was diagnosed algorithmically applying ICHD-II criteria. Disability was assessed as lost productive time by HALT index.

## Results

Age-standardized 1-year prevalence was 25.2% (95% CI: 23.9-27.4%; 10.6% definite, 14.6% probable migraine). Point prevalence (headache yesterday) was 2.7%. Prevalence was greater among females (31.6% vs 18.5%; OR=2.03 [95% CI: 1.64-2.50]) and in rural areas (28.9% vs 21.7%; OR=1.45 [95% CI: 1.16-1.82]). Prevalence peaked between 35-45 years in both genders. Median frequency was 24 days/year, with a sizeable minority (6.6%) reporting >60 days/year. Headache intensity was severe in 40%. Lost productive time correlated with attack frequency. The overall mean total was 3.7 ±6.1 days/3 months, representing a loss of 6.1% of productive days, of which 2.1 ±4.0 days/3 months were lost at home and 1.4 ±4.1 days/months were lost in the work place. Disability was higher among women and in rural areas.

## Conclusions

Migraine is highly prevalent in this part of India, and associated with substantial disability, especially among women and rural populations.

No conflict of interest.

